# Will you go the distance? A satisfaction survey of telemedicine in sexual medicine

**DOI:** 10.1093/sexmed/qfad060

**Published:** 2023-12-18

**Authors:** Gal Saffati, Taher Naeem, Jordan Kassab, Daniela Orozco Rendon, Charles Green, Larry I Liphsultz, Mohit Khera

**Affiliations:** Scott Department of Urology, Baylor College of Medicine, 7200 Cambridge Street, Houston, Texas, 77030, United States; Scott Department of Urology, Baylor College of Medicine, 7200 Cambridge Street, Houston, Texas, 77030, United States; Texas Tech University Health Sciences Center School of Medicine, 3601 4th Street, Lubbock, Texas 79430, United States; Scott Department of Urology, Baylor College of Medicine, 7200 Cambridge Street, Houston, Texas, 77030, United States; Department of Pediatrics, McGovern Medical School, University of Texas, 6431 Fannin Street, Houston, Texas 77030, United States; Scott Department of Urology, Baylor College of Medicine, 7200 Cambridge Street, Houston, Texas, 77030, United States; Scott Department of Urology, Baylor College of Medicine, 7200 Cambridge Street, Houston, Texas, 77030, United States

**Keywords:** hypogonadism, telehealth, sexual health, andrology

## Abstract

**Background:**

The success of telemedicine depends on patient satisfaction with the care that they receive, which is impacted by the ease of use of the technology, quality of the connection, and perceived effectiveness of care.

**Aim:**

The study sought to evaluate patient satisfaction with telemedicine services in a high-volume andrology clinic.

**Methods:**

We included all patients who had a telemedicine appointment between January 1, 2020, and August 22, 2022. Demographic information was gathered, and a satisfaction survey was conducted using REDCap software. Data were grouped into 2 age categories, with ≥50 years as the cutoff (19-50 years; >50 years). The data were analyzed according to age, distance from the patient’s home to our center, and survey responses. Pearson’s chi-square test and ordinal logistic regression analyses were performed.

**Outcomes:**

The main outcome is satisfaction with telemedicine in a men’s sexual health context.

**Results:**

A total of 4071 patients were identified based on attending a telemedicine visit. Hypogonadism was the most common diagnosis. Other diagnoses included erectile dysfunction, varicocele, Peyronie’s disease, vasectomy, and infertility. In total, 613 patients completed the survey, with a mean age of 56.6 years. Older patients were less likely to prefer telemedicine (odds ratio [OR], 0.55; 95% confidence interval [CI], 0.36-0.80; *P* < .001), less likely to agree to a video visit because of privacy concerns (OR, 0.51; 95% CI, 0.35-0.75; *P* < .001), and less likely to recommend a telemedicine visit compared with their younger counterparts (OR, 0.37; 95% CI, 0.27-0.51; *P* < .001). The median distance was 22.4 (interquartile range, 7.5-57.5) miles. However, there was no significant association between distance and patients’ likelihood of preferring telehealth visits, including reviews of outside laboratories and imaging (OR, 1; 95% CI, 0.99-1; *P* = .35), belief in the quality of care provided via video visits (OR, 0.99, CI 0.99-1; *P* = .25), and overall preference for telehealth visits (OR, 0.99; 95% CI, 0.99-1; *P* = .35).

**Clinical Implications:**

Healthcare providers should consider the age of patients when deciding to offer telemedicine while addressing privacy concerns to provide adequate reassurance to patients who may have concerns about the quality of care provided through telemedicine.

**Strengths and Limitations:**

Our study achieved a substantial sample size that reached statistical significance. Conducted at a single academic center, our study was constrained, possibly introducing biases related to the institution’s advanced telemedicine system. Geographic and diagnostic limitations could lead to regional biases, affecting the generalizability of the findings.

**Conclusion:**

Older patients exhibited a lower inclination toward preferring telemedicine, along with decreased odds of endorsing in-person visits.

## Introduction

Telemedicine is defined as the use of electronic communication technologies, such as videoconferencing and phone consultations, to provide remote clinical healthcare services. Before the COVID-19 pandemic, telemedicine was primarily used as a supplement to in-person care, allowing for increased access to specialty care in underserved areas.^1^ However, the pandemic has prompted a rapid expansion of telemedicine, with many healthcare providers shifting to virtual consultations as a primary means of medical care delivery.^2^

Telemedicine has been gaining popularity in the field of urology prior to the COVID-19 pandemic. However, there were several barriers to adoption, such as reimbursement issues, lack of resources and time, and liability concerns, according to a survey conducted by the American Urological Association’s Telemedicine Workgroup.^3^ Despite these barriers, a recent study using a state insurance claims database showed that urology was leading among surgical subspecialties in terms of telehealth conversions of encounters during the pandemic.^4^ According to the 2020 American Urological Association annual census data, 71.5% of urologists confirmed participating in telemedicine, a significant increase from 11.9% in the previous year.^3^

Moreover, telemedicine has the potential to especially benefit the field of sexual medicine, as it allows for the safe management of common male sexual dysfunctions such as erectile dysfunction, premature ejaculation, and hypogonadism through remote encounters.^5^ The COVID-19 pandemic has also highlighted the importance of telehealth in sexual medicine, as telemedicine encounters associated with male sexual medicine made up a significantly larger portion of outpatient practice.^6^

The success of telemedicine ultimately depends on the patient’s experience. While telehealth may be more convenient for providers with the ability to see more patients in a shorter amount of time, it is the patient’s satisfaction with the care as well as the quality of care that they receive that ultimately determines the success of telemedicine. Factors that can affect patient satisfaction with telemedicine include ease of use of the technology, quality of the video or audio connection, and perceived effectiveness of the care received. Ensuring that patients have positive experiences with telemedicine is crucial for its continued success. Therefore, we sought to evaluate patient satisfaction with telemedicine services at our high-volume andrology clinic.

## Methods

An institutional review board–approved study was conducted to evaluate the telemedicine experience among male patients at our center. A query of the EPIC electronic medical record system was performed to identify all male patients who had at least 1 telemedicine appointment between January 1, 2020, and August 22, 2022. Basic demographic information, including age, race, encounter date, and International Classification of Diseases–Tenth Revision diagnosis codes, was also collected. Only patients seen by 2 high-volume andrologists at our center were included. A satisfaction survey, utilizing the REDCap (Research Electronic Data Capture) software, was administered to the eligible patients. The survey consisted of 5 sections, addressing basic information, purpose of visit, relationship and communication, quality of care, and satisfaction, and included a total of 17 questions. Sixteen of the questions were multiple choice, while 1 question was open-ended, seeking feedback on participants’ willingness to consider a video visit in the future. The full survey is included as supplemental material (Appendix 1). Data collection occurred from October 2022 to December 2022. To further analyze the data, respondents were subdivided by age, with a cutoff of 50 years of age, into 2 groups for comparison (19-50 years of age; >50 years of age). For each relevant question, a cross-tabulation between age group and responses was made, with subsequent Pearson’s chi-square test. Likewise, when using age as a continuous variable, a proportional odds logistic regression analysis was made while we used the dichotomized age groups as a predictor to get a measure of association. Finally, we calculated the distance in miles from the patient’s registered home address to our center and later compared it with their recorded responses to measure any relation with distance and satisfaction.

## Results

### Study population

A total of 4071 patients who had at least 1 telemedicine appointment between January 1, 2020, and August 22, 2022, were identified. Of these, 158 (3.8%) were Asian, 297 (7.3%) were Black or African American, 239 (5.8%) were Hispanic, 2700 (66.3%) were White, and 230 (5.6%) identified as other. A total of 447 (10.9%) patients had no recorded races. A total of 3121 (76.6%) patients had an office visit via video, while 950 (23.3%) visits were conducted over the telephone. The mean age of the study population was 50.1 years, with variation among racial groups. Asian participants had a mean age of 43.7 years, Black or African American participants had a mean age of 51.1 years, Hispanic participants had a mean age of 42.2 years, and White participants had a mean age of 51.9 years. The study population was characterized by a wide range of diagnoses. The most common diagnosis was hypogonadism, which accounted for 89.1% of patients. This was followed by erectile dysfunction, with 4.6%. In addition, varicocele accounted for 2.2% of the patients, and Peyronie’s disease accounted for 2.2% of the patients. Vasectomy accounted for 1.2% of the patients, while Infertility accounted for 0.8% of the patients.

## Responses

A total of 613 patients completed the survey, with a mean age of 56.6 years. Respondent characteristics are summarized in Table 1. All survey responses are included in Appendix 1, while relevant questions and answers are summarized in Table 2.

**Table 1:** Demographic characteristics of survey respondents.

**Age**	
<50 y	201 (32.8)
≥50 y	412 (67.2)
**Race/ethnicity**	
American Indian or Alaska Native	2 (0.3)
Asian Indian	1 (0.1)
Black or African American	23 (3.7)
Hispanic	22 (3.5)
Other Asian	8 (1.3)
Other Pacific Islander	1 (0.1)
White	487 (79.4)
Declined to answer racial/ethnic identification	40 (6.5)
None of the above	29 (4.7)
**Diagnosis**	
Hypogonadism	313 (51.1)
Erectile dysfunction and hypogonadism	134 (21.9)
Erectile dysfunction	64 (10.4)
Infertility	32 (5.2)
Don’t know or don’t remember diagnosis	15 (2.5)
Infertility and hypogonadism	14 (2.3)
Peyronie’s disease	13 (2.1)
**Subjective overall general state of health**	
Fully active without restrictions	208 (34.0)
Minimal difficulty engaging in most activities	98 (16.0)
Somewhat difficult to engage in some activities	158 (25.7)
Very difficult for me to engage in some activities	80 (13.0)
Very difficult for me to engage in most activities	69 (11.2)

Values are n (%).

**Table 2:** Relevant survey responses stratified by age.

Question	Survey sample, % of subgroup	
	<50 years of age	≥50 years of age
1. How likely would you be to meet with your Urology provider via video call from your home when you do NOT need new labs/imaging, your physician can review outside labs/imaging, and no physical exam/procedure is needed?		
*Very unlikely*	1.0	4.4
*Somewhat unlikely*	1.5	5.3
*Equally likely or unlikely*	4.5	6.3
*Somewhat likely*	13.9	15.3
*Very likely*	79.1	68.7
7. I believe the provider is able to do his/her job adequately despite not being able to conduct a physical exam at every appointment.		
*Strongly disagree*	2.0	4.1
*Moderately disagree*	1.0	7.3
*Equally agree and disagree*	8.5	13.6
*Moderately agree*	18.4	28.2
*Strongly agree*	70.1	46.8
8. I feel confident that video conferencing communications are private and secure.		
*Strongly disagree*	0.5	3.6
*Moderately disagree*	0.5	4.4
*Equally agree and disagree*	5.0	8.5
*Moderately agree*	18.9	21.1
*Strongly agree*	75.1	62.4
9. I would prefer to see a provider over video rather than travel to a physical appointment.		
*Strongly disagree*	5.5	14.1
*Moderately disagree*	7.0	16.0
*Equally agree and disagree*	26.4	25.5
*Moderately agree*	13.9	17.0
*Strongly agree*	47.3	27.4
10. I believe I will get the same quality of care via video visit as I would during an in-person visit.		
*Strongly disagree*	3.0	11.2
*Moderately disagree*	7.0	16.0
*Equally agree and disagree*	17.9	19.2
*Moderately agree*	16.9	23.1
*Strongly agree*	55.2	30.6
12. How satisfied are you with the telehealth visits you have had at our center?		
*Very dissatisfied*	1.5	4.9
*Somewhat dissatisfied*	1.5	2.7
*Equally satisfied and dissatisfied*	10.4	16.5
*Somewhat satisfied*	13.4	15.8
*Very satisfied*	73.1	60.2
14. If you had to do it again, would you prefer a telehealth visit or an in-person visit?		
*Telehealth*	75.1	55.1
*In-person*	24.9	44.9
15. How likely are you to recommend a telehealth visit with our center over an in-person visit to friends or family?		
*Very unlikely*	0.5	9.5
*Somewhat unlikely*	4.5	9.0
*Equally likely or unlikely*	12.4	22.6
*Somewhat likely*	22.4	19.9
*Very likely*	60.2	39.1

In terms of **preference for telemedicine** over in-person visits, 61.2% of younger patients preferred telemedicine compared with 44.4% of older patients. Overall, it was found that older patients were significantly less likely to prefer telemedicine visits (odds ratio [OR], 0.43; 95% confidence interval [CI], 0.31-0.58; *P* < .001). This was further supported when we stratified by age, and a downward trend in preference for telemedicine was observed, with older patients being less likely to prefer telemedicine to their younger counterparts OR 0.55; 95% CI, 0.36-0.80; *P* < .001). With each year of age, the odds of a patient preferring telemedicine decreased (OR, 0.97; 95% CI, 0.96-0.98; *P* < .001). Older patients were less likely to recommend a telemedicine visit than younger patients (OR, 0.37; 95% CI, 0.26-0.51; *P* < .001). Younger patients were found to be more satisfied with telemedicine visits than older patients (OR, 1.85; 95% CI 1.32-2.70; *P* < .001). Furthermore, with each additional year of age, the odds of endorsing an in-person visit increased (OR, 1.03; 95% CI, 1.01-1.04; *P* < .001).

Delving into specific concerns that patients had with telemedicine, there were concerns among older patients about the quality, security, and limitations of telemedicine. When assessing the patients’ beliefs about the ability of providers to perform their job competently without physical examinations, older patients disagreed that providers could perform their job correctly with telemedicine appointments (*P* < .001) Regarding privacy and security concerns, younger patients were more confident that video visits were private and secure than older patients (94% vs 83%). Older patients were less likely to agree to a video visit because of privacy concerns (OR, 0.51; 95% CI, 0.35-0.75; *P* < .001), and with each additional year of age, the agreement with the question decreased (OR, 0.98; 95% CI, 0.96-0.99; *P* < .001). In terms of quality of care provided and patient satisfaction, older patients were also less likely to agree that the quality of care would be the same between video visits and in-person appointments than younger patients (OR, 0.37; 95% CI, 0.27-0.50; *P* < .001).

## Distance

The median distance was 22.4 (interquartile range, 7.5-57.5) miles. When evaluating the relationship between distance and patient satisfaction with telehealth visits, we found no significant association between distance and the likelihood of a patient preferring telehealth visits for review of outside labs and imaging without the need for new tests, physical examinations, or procedures (OR, 1; 95% CI, 0.99-1; *P* < .01). There was also no significant decrease in belief in the provider’s ability to perform their job adequately despite the absence of a physical exam at every appointment (OR, 0.99, CI 0.99-1; *P* < .01) or confidence in the privacy and security of video visits (OR, 0.99; 95% CI, 0.99-1; *P* < .01). There was no preference for telehealth visits over traveling for in-person visits (OR, 1.33; 95% CI, 0.99-1.8; *P* < .01) related to distance. There was no relationship between the belief in the same quality of care via video visit as in-person visit (OR, 1; 95% CI, 0.99-1; *P* < .01) that was related to distance. Last, the overall preference for telehealth visits (OR, 0.99; 95% CI, 0.99-1; *P* < .01) was not significantly impacted by distance.

## Discussion

The inclusion of telemedicine across healthcare providers has proven to be critical, allowing patients to receive medical consultations, diagnoses, and treatment from clinicians located in other areas without the need to travel long distances. This is especially crucial for patients with chronic conditions that require frequent medical attention. Urology, as a telemedicine subspecialty, has potentially even more useful implications because of the high prevalence of chronic disorders experienced by both male and female patients.^7^ Additionally, as specialty care is perceived as less accessible due to its high cost of service, telemedicine can help remediate this barrier by reducing the time and cost associated with patient travel.^8^ The study underscores the potential advantages of telemedicine in urology.

Our findings explores several factors that shape patients’ inclinations toward telemedicine, such as age and privacy concerns. Specifically, the results indicated that older patients exhibited a reduced preference for telemedicine and were less likely to recommend it to others. Furthermore, our data revealed emerging concerns regarding privacy as a significant impediment to telemedicine adoption. According to the results, patients who had to travel long distances to reach the healthcare center reported higher satisfaction levels with telehealth visits. This observation could assist healthcare providers in identifying patients who are likely to gain the most from telemedicine and refining their services to better suit their requirements. These insights can help healthcare providers to develop more effective strategies for integrating telemedicine into clinical practice and improving patient satisfaction. The implications of this study are as follows: healthcare providers should consider the age of patients when deciding to offer telemedicine while addressing privacy concerns to provide adequate reassurance to patients who may have concerns about the quality of care provided through telemedicine.

Although the sample size was quite substantial, encompassing hundreds of patient inputs, a low response rate was observed in our study (15.1%). This lack of response may be due to the sensitive nature of urology, which limits what patients feel comfortable sharing online. Additionally, because this study was conducted at a single academic center, it limited the range of patients that we were able to assess. This can lead to biases that stem from the institution’s ability to perform telemedicine adequately. The institution in which this study was conducted has a well-established protocol for telemedicine in place that may make it a more viable option than in other institutions where the system may be less developed. Thus, the results could be reflective of this iteration of telemedicine and not necessarily reflect just telemedicine as its own distinct service. There may be regional biases associated with our data because of this limitation in the geographic and diagnostic scope; thus, these results may not be generalizable to the general population. However, we believe that this potential drawback may be mitigated by a large sample size, including subjects with diverse backgrounds, and varying diagnosis within sexual medicine. To build on these data, future studies should include a larger sample size and multiple centers to increase the generalizability of the results and ensure reliability and external validity.^9^ Another potential source of bias stems from the survey only being distributed among patients who had done a telemedicine visit, which could introduce that bias that these patients would be more likely to prefer a telemedicine visit than the general population. Further, it would be of note to investigate other potential confounding variables, such as socioeconomic status, education level, previous experience with telemedicine, and health status, as indicated by the Charlson Comorbidity Index. Because these factors are highly variable among individuals, they may lead to further divided results. Clinicians can provide more comprehensive recommendations in their counseling by knowing what various populations are likely to prefer.

This study highlights the importance of further research to refine our understanding of the relationship between telemedicine, patient satisfaction, and exceptional healthcare, particularly regarding the influence of socioeconomic status, education level, and overall health status on reported telehealth satisfaction in specific patient subpopulations. Moreover, future studies could explore the potential of telemedicine in the diagnosis and management of specific urological conditions, ultimately providing valuable insights into the benefits of telemedicine in the field of urology. Furthermore, this study has implications beyond the realm of urology; by expanding into different medical specialties, we can uncover telemedicine’s transformative impact across healthcare to have a more comprehensive scope of its relationship with patient satisfaction. As telemedicine continues to evolve, further research and development in this area could have a substantial impact on the healthcare industry, improving patient outcomes and ultimately contributing to a healthier and more connected society.

## Conclusion

Patient preferences and concerns when introducing telemedicine services, particularly for the elderly population, are critically important in ensuring the ultimate success of telemedicine implementation. This knowledge can help healthcare providers tailor their services to meet the needs of their patients and enhance the adoption of telemedicine.

## Supplementary Material

STROBE_Telehealth_qfad060

## Figures and Tables

**Appendix 1:** Survey responses

Question	Options	N (%)
0. Which statement best describes your overall general state of health?	1 - It is VERY DIFFICULT for me to engage in MOST activities	15 (2.4)
	2 - It is VERY DIFFICULT for me to engage in SOME activities	22 (3.6)
	3 - It is SOMEWHAT DIFFICULT for me to engage in SOME activities	87 (14.2)
	4 - I am able to engage in most activities with MINIMAL difficulty	180 (29.3)
	5 - I am fully active without any restrictions	309 (50.4)
1. How likely would you be to meet with your Urology provider via video call from your home when you do NOT need new labs/imaging, your physician can review outside labs/imaging, and no physical exam/procedure is needed?	1 - Very unlikely	20 (3.3)
	2 - Somewhat unlikely	25 (4.1)
	3 - Equally likely and unlikely	35 (5.7)
	4 - Somewhat likely	91 (14.8)
	5 – Very likely	442 (72.1)
2. Undergoing an initial clinic visit with a new urology provider?	1 - Strongly Disagree	111 (18.1)
	2 - Moderately Disagree	115 (18.7)
	3 - Equally Agree and Disagree	100 (16.3)
	4 - Moderately Agree	139 (22.7)
	5 - Strongly Agree	148 (24.1)
3. Discussing new symptoms and/or concerns?	1 - Strongly Disagree	36 (5.8)
	2 - Moderately Disagree	47 (7.6)
	3 - Equally Agree and Disagree	53 (8.6)
	4 - Moderately Agree	149 (24.3)
	5 - Strongly Agree	328 (53.5)
4. Discussing diagnosis, treatment, and/or follow-up recommendations?	1 - Strongly Disagree	34 (5.6)
	2 - Moderately Disagree	39 (6.4)
	3 - Equally Agree and Disagree	47 (7.7)
	4 - Moderately Agree	123 (20)
	5 - Strongly Agree	370 (60.3)
5. Reviewing imaging and laboratory test findings?	1 - Strongly Disagree	30 (4.9)
	2 - Moderately Disagree	26 (4.2)
	3 - Equally Agree and Disagree	46 (7.5)
	4 - Moderately Agree	131 (21.3)
	5 - Strongly Agree	380 (62)
6. Completing postoperative follow-up?	1 - Strongly Disagree	58 (9.5)
	2 - Moderately Disagree	71 (11.6)
	3 - Equally Agree and Disagree	71 (11.6)
	4 - Moderately Agree	133 (21.7)
	5 - Strongly Agree	280 (45.7)
7. I believe the provider is able to do his/her job adequately despite not being able to conduct a physical exam at every appointment.	1 - Strongly Disagree	21 (3.4)
	2 - Moderately Disagree	32 (5.2)
	3 - Equally Agree and Disagree	73 (11.9)
	4 - Moderately Agree	153 (24.9)
	5 - Strongly Agree	334 (54.5)
8. I feel confident that video conferencing communications are private and secure.	1 - Strongly Disagree	16 (2.6)
	2 - Moderately Disagree	19 (3.1)
	3 - Equally Agree and Disagree	45 (7.3)
	4 - Moderately Agree	125 (20.4)
	5 - Strongly Agree	408 (66.5)
9. I would prefer to see a provider over video rather than travel to a physical appointment.	1 - Strongly Disagree	69 (11.2)
	2 - Moderately Disagree	80 (13)
	3 - Equally Agree and Disagree	158 (25.8)
	4 - Moderately Agree	98 (16.0)
	5 - Strongly Agree	208 (34.0)
10. I believe I will get the same quality of care via video visit as I would during an in-person visit.	1 - Strongly Disagree	52 (8.5)
	2 - Moderately Disagree	80 (13.1)
	3 - Equally Agree and Disagree	115 (18.8)
	4 - Moderately Agree	129 (21.0)
	5 - Strongly Agree	237 (38.7)
12. How satisfied are you with the telehealth visits you have had at our center?	1 - Strongly Disagree	23 (3.8)
	2 - Moderately Disagree	14 (2.3)
	3 - Equally Agree and Disagree	89 (14.5)
	4 - Moderately Agree	92 (15.0)
	5 - Strongly Agree	395 (64.4)
13. Did your video visit with your Baylor College of Medicine Urology provider start on time?	1 - Yes	475 (77.5)
	2 - No	138 (22.5)
14. If you had to do it again, would you prefer a telehealth visit or an in-person visit?	1 - Telehealth	378 (61.7)
	2 - In-person	235 (38.3)
15. How likely are you to recommend a telehealth visit with Baylor College of Medicine Urology over an in-person visit to friends or family?	1 - Very unlikely	40(6.5)
	2 - Somewhat unlikely	46 (7.5)
	3 - Equally likely or unlikely	118(19.2)
	4 - Somewhat likely	127(20.7)
	5 - Very likely	282(46.0)
