# Supplementary material for: Will you go the distance? A satisfaction survey of telemedicine in sexual medicine
Source: Sex Med. 2023 Dec 18;11(6):qfad060. doi: 10.1093/sexmed/qfad060 (PMC10727694; doi:10.1093/sexmed/qfad060)
Supplement: STROBE_Telehealth_qfad060 [file strobe_telehealth_qfad060.docx]

STROBE Statement—checklist of items that should be included in reports of observational studies

|  | Item No. | Recommendation | Page  No. | Relevant text from manuscript |
| --- | --- | --- | --- | --- |
| **Title and abstract** | 1 | (*a*) Indicate the study’s design with a commonly used term in the title or the abstract | 1, 2 | *“…a satisfaction survey…”* |
|  |  | (*b*) Provide in the abstract an informative and balanced summary of what was done and what was found | 2 | See Methods and Results |
| Introduction | | | |  |
| Background/rationale | 2 | Explain the scientific background and rationale for the investigation being reported | 3 | *“…the importance of telehealth in sexual medicine, as telemedicine encounters associated with male sexual medicine made up a significantly larger portion of outpatient practice. The success of telemedicine ultimately depends on the patient's experience. While telehealth may be more convenient for providers with the ability to see more patients in a shorter amount of time, it is the patient's satisfaction with the care…”* |
| Objectives | 3 | State specific objectives, including any prespecified hypotheses | 4 | *“…to evaluate patient satisfaction with telemedicine services at our high-volume andrology clinic…”* |
| Methods | | | |  |
| Study design | 4 | Present key elements of study design early in the paper | 4 |  |
| Setting | 5 | Describe the setting, locations, and relevant dates, including periods of recruitment, exposure, follow-up, and data collection | 4 | *“An institutional review board-approved study was conducted to evaluate the telemedicine experience among male patients at our center. A query of the EPIC electronic medical record system was performed to identify all male patients who had at least one telemedicine appointment between January 1, 2020, and August 22, 2022…”* |
| Participants | 6 | (*a*) *Cohort study*—Give the eligibility criteria, and the sources and methods of selection of participants. Describe methods of follow-up  *Case-control study*—Give the eligibility criteria, and the sources and methods of case ascertainment and control selection. Give the rationale for the choice of cases and controls  *Cross-sectional study*—Give the eligibility criteria, and the sources and methods of selection of participants | 4 | *“all male patients who had at least one telemedicine appointment between January 1, 2020, and August 22, 2022”* |
|  |  | (*b*) *Cohort study*—For matched studies, give matching criteria and number of exposed and unexposed  *Case-control study*—For matched studies, give matching criteria and the number of controls per case |  |  |
| Variables | 7 | Clearly define all outcomes, exposures, predictors, potential confounders, and effect modifiers. Give diagnostic criteria, if applicable | 4 | See Methods |
| Data sources/ measurement | 8* | For each variable of interest, give sources of data and details of methods of assessment (measurement). Describe comparability of assessment methods if there is more than one group | *4* |  |
| Bias | 9 | Describe any efforts to address potential sources of bias | 4 |  |
| Study size | 10 | Explain how the study size was arrived at | 4 |  |

Continued on next page

| Quantitative variables | 11 | Explain how quantitative variables were handled in the analyses. If applicable, describe which groupings were chosen and why | 4 | See Methods |
| --- | --- | --- | --- | --- |
| Statistical methods | 12 | (*a*) Describe all statistical methods, including those used to control for confounding | 4 |  |
|  |  | (*b*) Describe any methods used to examine subgroups and interactions | 4 |  |
|  |  | (*c*) Explain how missing data were addressed | 4 |  |
|  |  | (*d*) *Cohort study*—If applicable, explain how loss to follow-up was addressed  *Case-control study*—If applicable, explain how matching of cases and controls was addressed  *Cross-sectional study*—If applicable, describe analytical methods taking account of sampling strategy | 4 |  |
|  |  | (*e*) Describe any sensitivity analyses | NA |  |
| Results | | | | |
| Participants | 13* | (a) Report numbers of individuals at each stage of study—eg numbers potentially eligible, examined for eligibility, confirmed eligible, included in the study, completing follow-up, and analysed | 5 | A total of 4,071 patients who had at least one telemedicine appointment between January 1, 2020, and August 22, 2022, were identified. Of these, 2700 (66.3%) were white, 297 (7.3%) were Black or African American, 239 (5.8%) were Hispanic, 158 (3.8%) were Asian, and 230 (5.6%) identified as other. A total of 447 (10.9%) patients had no recorded races. A total of 3,121 patients (76.6%) had an office visit via video, while 950 (23.3%) visits were conducted over the telephone. |
|  |  | (b) Give reasons for non-participation at each stage | NA |  |
|  |  | (c) Consider use of a flow diagram | NA |  |
| Descriptive data | 14* | (a) Give characteristics of study participants (eg demographic, clinical, social) and information on exposures and potential confounders | 5 | The mean age of the study population was 50.1 years, with variation among racial groups. White participants had a mean age of 51.9 years; Black or African American participants of 51.1 years; Hispanic participants of 42.2 years; and Asian participants of 43.7 years. The study population was characterized by a wide range of diagnoses. The most common diagnosis was hypogonadism, which accounted for 89.1% of patients. This was followed by erectile dysfunction with 4.6%. In addition, varicocele accounted for 2.2% of the patients, and Peyronie’s accounted for 2.2% of the patients. Vasectomy accounted for 1.2% of the patients, while Infertility accounted for 0.8% of the patients. |
|  |  | (b) Indicate number of participants with missing data for each variable of interest | 5 | A total of 447 (10.9%) patients had no recorded races. |
|  |  | (c) *Cohort study*—Summarise follow-up time (eg, average and total amount) |  |  |
| Outcome data | 15* | *Cohort study*—Report numbers of outcome events or summary measures over time |  |  |
|  |  | *Case-control study—*Report numbers in each exposure category, or summary measures of exposure |  |  |
|  |  | *Cross-sectional study—*Report numbers of outcome events or summary measures | *5-6* | *A total of 613 patients completed the survey* |
| Main results | 16 | (*a*) Give unadjusted estimates and, if applicable, confounder-adjusted estimates and their precision (eg, 95% confidence interval). Make clear which confounders were adjusted for and why they were included | 5-7 | See Results |
|  |  | (*b*) Report category boundaries when continuous variables were categorized | 5-7 | See Results |
|  |  | (*c*) If relevant, consider translating estimates of relative risk into absolute risk for a meaningful time period |  |  |

Continued on next page

| Other analyses | 17 | Report other analyses done—eg analyses of subgroups and interactions, and sensitivity analyses | 5-7 | See Results |
| --- | --- | --- | --- | --- |
| Discussion | | | | |
| Key results | 18 | Summarise key results with reference to study objectives | 7 | See Discussion |
| Limitations | 19 | Discuss limitations of the study, taking into account sources of potential bias or imprecision. Discuss both direction and magnitude of any potential bias | 8 | See Discussion |
| Interpretation | 20 | Give a cautious overall interpretation of results considering objectives, limitations, multiplicity of analyses, results from similar studies, and other relevant evidence | 8 | See Discussion |
| Generalisability | 21 | Discuss the generalisability (external validity) of the study results | 8 | See Discussion |
| Other information | |  | | |
| Funding | 22 | Give the source of funding and the role of the funders for the present study and, if applicable, for the original study on which the present article is based | 1 |  |

*Give information separately for cases and controls in case-control studies and, if applicable, for exposed and unexposed groups in cohort and cross-sectional studies.

**Note:** An Explanation and Elaboration article discusses each checklist item and gives methodological background and published examples of transparent reporting. The STROBE checklist is best used in conjunction with this article (freely available on the Web sites of PLoS Medicine at http://www.plosmedicine.org/, Annals of Internal Medicine at http://www.annals.org/, and Epidemiology at http://www.epidem.com/). Information on the STROBE Initiative is available at www.strobe-statement.org.
